# Finite Element Modelling of Single Cell Based on Atomic Force Microscope Indentation Method

**DOI:** 10.1155/2019/7895061

**Published:** 2019-12-20

**Authors:** Lili Wang, Li Wang, Limeng Xu, Weiyi Chen

**Affiliations:** ^1^Shanxi Key Laboratory of Material Strength & Structural Impact, College of Biomedical Engineering, Taiyuan University of Technology, Taiyuan 030024, China; ^2^National Demonstration Center for Experimental Mechanics Education, Taiyuan University of Technology, Taiyuan 030024, China

## Abstract

The stiffness of cells, especially cancer cells, is a key mechanical property that is closely associated with their biomechanical functions, such as the mechanotransduction and the metastasis mechanisms of cancer cells. In light of the low survival rate of single cells and measurement uncertainty, the finite element method (FEM) was used to quantify the deformations and predict the stiffness of single cells. To study the effect of the cell components on overall stiffness, two new FEM models were proposed based on the atomic force microscopy (AFM) indentation method. The geometric sizes of the FEM models were determined by AFM topography images, and the validity of the FEM models was verified by comparison with experimental data. The effect of the intermediate filaments (IFs) and material properties of the cellular continuum components on the overall stiffness were investigated. The experimental results showed that the stiffness of cancer cells has apparent positional differences. The FEM simulation results show that IFs contribute only slightly to the overall stiffness within 10% strain, although they can transfer forces directly from the membrane to the nucleus. The cytoskeleton (CSK) is the major mechanical component of a cell. Furthermore, parameter studies revealed that the material properties (thickness and elasticity) of the continuum have a significant influence on the overall cellular stiffness while Poisson's ratio has less of an influence on the overall cellular stiffness. The proposed FEM models can determine the contribution of the major components of the cells to the overall cellular stiffness and provide insights for understanding the response of cells to the external mechanical stimuli and studying the corresponding mechanical mechanisms and cell biomechanics.

## 1. Introduction

Cell stiffness has an important influence on the cell biomechanical functions of cells and the mechanisms of mechanotransduction, such as cell motility, pathophysiology, and metastasis mechanisms of cancer cells [1]. In general, cell stiffness can be determined by optical/magnetic tweezing [2, 3], micropipette aspiration [4], and atomic force microscopy (AFM) indentation [5], but AFM is widely used for testing and understanding the mechanical properties of the living cells due to the accurate control of force and location in AFM. For example, Cross et al. [6] used the AFM indentation methods to measure Young's modulus of live metastatic cancer cells taken from pleural effusions of patients. Li et al. [7] and Nikkhah et al. [8] measured the elasticity of malignant breast epithelial cells. Hayashi and Iwata [9] used the same technique to study the stiffness of HeLa cells and End1/E6E7 at different locations.

However, the mechanical properties such as the stiffness, elasticity, and viscoelasticity of cells were studied mainly through experimental methods. Considering that the survival rate of a single cell is low and the measurement is uncertain, a FEM model of a single cell can quantify the deformations of the cell and predict the stiffness of the cell. FEM modelling of cells is favoured by researchers in the field of cellular mechanics. For example, Chen and Lu [10] conducted a 2D model for AFM nanoindentation on chondrocytes by assuming the cells to be a homogeneous viscoelastic material. Baaijens et al. [11] developed a 2D nonstructural model of a chondrocyte with homogenous elasticity to achieve the cell's response subjected to micropipette aspiration. Caille et al. [12] created a 2D structural model of an endothelial cell with homogeneous incompressible hyperelasticity to predict its mechanical properties under compression. Unnikrishnan et al. [13] applied a finite element model of an adherent cell to numerically simulate the AFM indentation. The above researchers did not consider the role of the cytoskeleton (CSK). McGarry and Prendergast [14] carried out a 3D FEM model of an adherent cell to describe the nonlinear structural properties and ascertain the influence of the various cellular components on cellular stability. Unfortunately, they did not consider the effect of intermediate filaments (IFs). Furthermore, the FEM models have not been verified with experimental results. Because IFs play a crucial role in cell mechanics based on recent evidence and their role is closely related to that of disease [15], it is necessary to study the mechanical properties of IFs in living cells. Some researchers [16–19] have also emphasized that IFs are an important regulator of cell stiffness, as determined through experimental methods; for example, Janmey et al. [20] and Wang and Stamenović [21] showed that IFs have an important effect on cellular rigidity only when the strain is above 20%. However, the effect of IFs on cellular rigidity in small deformation conditions (approximately below 10% strain) is still unknown.

In this study, two new FEM models of single cells were constructed to study the effect of IFs on overall cellular stiffness based on the AFM indentation method. The FEM models are composed of cell membrane, a nucleus, a cytoplasm, and a CSK, and their geometric sizes were determined through AFM topography images. The proposed FEM models were validated by comparing the simulated results with the AFM experimental results at two different locations. To investigate the influence of the cellular components on the overall stiffness, parameter analysis was performed. Therefore, the development model can provide a deeper understanding of the response of the cells to the external mechanical stimuli and mechanotransduction.

## 2. Methods

### 2.1. Cell Preparation and AFM Indentation Tests

HeLa cells (human cervical cancer cells) were selected for the present study because they had been widely used as a representative of cancer cells [9, 22, 23]. The HeLa cells were seeded on sterilized plastic dishes and cultured in Dulbecco's Modified Eagle's Medium (DMEM) with 10% (V/V) foetal bovine serum (FBS) and 1% (V/V) penicillin-streptomycin. Promptly after seeding, the cells were kept at 37°C in a 5% CO_2_ incubator for 24 h. Then, the dishes were rinsed with phosphate buffer solution (PBS) and 1 ml DMEM to remove dead and loosely attached cells just before AFM measurement.

**Figure 1:**
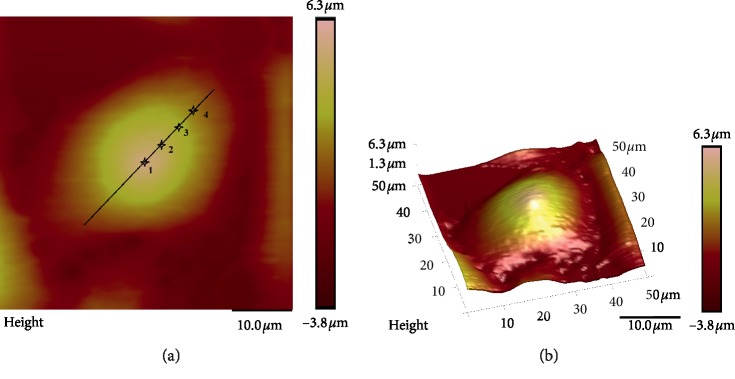
Schematic diagram of the locations of the AFM tip and 3D structure of the HeLa cell: point 1 is at the cell centre; point 2 is 1/4 of the major diameter from the cell centre; point 3 is 1/2 of the major diameter from the cell centre; point 4 is 3/4 of the major diameter from the cell centre. (a) Indenting locations and 2D structure. (b) 3D structure.

For the AFM indentation tests, the spring constant of the cantilever is 0.03 N/m, and a conical indenter with a half-opening of 18° and a tip radius of 20 nm was used. Indentation tests were performed at four different positions, which were at the centre (point 1), 1/4 (point 2), 1/2 (point 3), and 3/4 (point 4) of the major diameter from the cell centre; three tests were conducted at each position, as shown in Figure 1. The results were determined using the average of the experimental data. The geometric sizes of the cells were determined using AFM topography images because the AFM can provide detailed information about the topography of the cytoplasm membrane [24]. The elastic moduli of the cells at different locations were obtained by fitting force (F)-indentation (*δ*) data using the Hertzian and Sneddon formula that relates the indentation force and indentation depth, which is expressed as [25]

(1) $$\begin{array}{c} F=\frac{2E\delta^{2}}{\pi \left(1-\nu^{2}\right)}\tan\left(\alpha \right), \end{array}$$

where *E* is the cellular elastic modulus, *F* is the reaction force at the indenter tip, *α* is a half-opening angle of the indenter tip, *v* is Poisson's ratio, and *δ* is the indentation depth.

### 2.2. FEM Model

**Figure 2:**
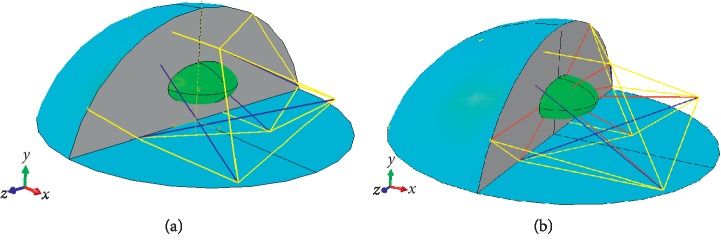
Schematic diagram of the 3D FEM model of a single cell: the cell consists of a membrane (cyan), cytoplasm (grey), nucleus (green), and cytoskeleton (yellow represents MFs, blue represents MTs, and red represents IFs). (a) CSK without IFs. (b) CSK including IFs.

To study the contribution of IFs to the overall cellular stiffness, two FEM models of single cells composed of the major cellular components, including the membrane, cytoplasm, nucleus and CSK, are established. The CSK of one model is composed of microtubules (MTs) and microfilaments (MFs) (Figure 2(a)), and the CSK of the other model is composed of MTs, MFs, and IFs (Figure 2(b)). Based on previous studies [26], the FEM model of single cells is approximated as a spherical cap with a contact radius of 12 *μ*m and a height of 8.9 *μ*m according to the AFM optical images, and the nucleus is modelled as an ellipsoid with a major axis of 6 *μ*m. The minor axis is 3 *μ*m [27], and its centre lies at the centroid of the cell by using the formulation expressed as

(2) $$\begin{array}{c} y=\frac{\int_{A}y\text{d}V}{V}=\frac{\pi \int_{R-h}^{R}y\left(R^{2}-y^{2}\right)\text{d}y}{\left(\pi /3\right)\left(3R-h\right)h^{2}}=\frac{3r_{0}^{4}}{2h\left(3r_{0}^{2}+h^{2}\right)}-\frac{r_{0}^{2}-h^{2}}{2h}. \end{array}$$

### 2.3. Boundary Conditions and Material Properties

The geometries of the models are created using UG NX 10.0 (Unigraphics NX 10.0) and then imported into the commercial finite element package ABAQUS (standard version 6.13, SIMULIA, Germany) for simulations and analysis. In the modelling, although most components of the cell exhibit approximately nonlinear constitutive behaviour; the membrane, cytoplasm, and nucleus are treated as isotropic linearly elastic continua, and the CSK is assumed to be a 12-node tensegrity structure. In particular, the cell membrane is assumed to be a shell, the cytoplasm is filled by the cell, and the CSK is treated as a truss embedded in the model. In the tensegrity structure, the cables represent the MFs and IFs, and the struts represent the MTs. Only one truss element is used. The membrane is meshed with shell elements; the element type is S4R, and the total number of elements is 178,180. The cytoplasm is meshed with hexahedron elements; the element type is C3D8R, and the total number of elements is 73,404. The nucleus is meshed with tetrahedron elements; the element type is C3D4, and the total number of elements is 13,273. The material parameters are taken from the literature listed in Table 1.

The cell is indented at a load velocity of 1 *μ*m/*μ*s with a targeted indentation depth of 1 *μ*m. The basal surface of the cell is constrained in all directions. Two indenting locations (points 1 and 4) are chosen to verify the rationality and validity of the models. However, parametric analysis was only performed at the cell centre (point 1). Due to the usage of a conical indenter in the AFM tests and irrespective of the variation in the area of the contact surface during simulation, the point load is selected.

## 3. Results

### 3.1. Experimental Results

**Figure 3:**
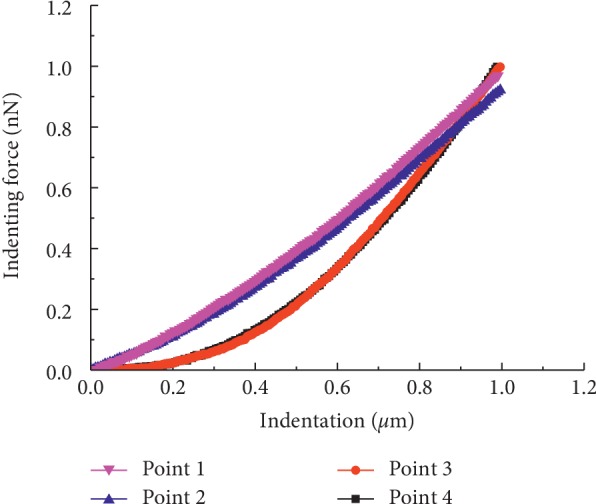
Force-indentation curves from the AFM indentation experiment at four different locations.

There are four indenting force-indentation curves, as shown in Figure 3, and each curve represents one indenting location. The elastic modulus of the cells was calculated using equation (1); the average at point 1 is 2.57 kPa, the average at point 2 is 2.13 kPa, the average at point 3 is 4.86 kPa, and the average at point 4 is 4.58 kPa. The experimental results suggested that the stiffness of the cancer cells has apparent positional differences, similar to previously reported results [25, 32].

### 3.2. Finite Element Modelling Results

#### 3.2.1. Model Verification

**Figure 4:**
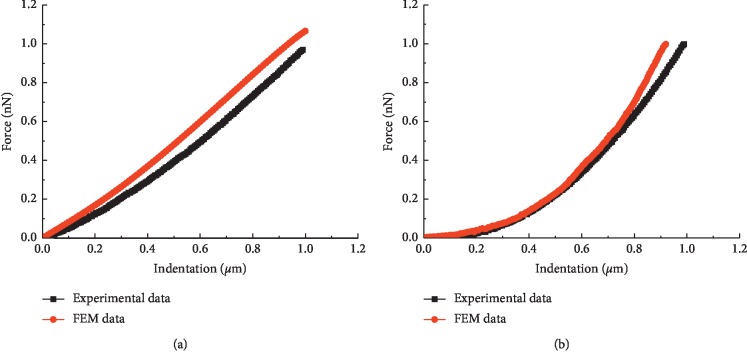
Force-indentation curves at different acting locations (not including IFs). Indenting location: (a) point 1; (b) point 4.

The properties of points 2 and 3 are very similar to those of points 1 and 4, respectively (Figure 3). The developed FEM models are verified by selecting two indentation points, point 1 (at the cell centre) and point 4 (3/4 of the major diameter from the cell centre), which are chosen according to the experimental measurement positions. The force-indentation curves are demonstrated in Figure 4, where the black lines represent the experimental data and the red lines represent the simulation data.

The results show that the simulation force-indentation curves agree well with the experimental data. Therefore, the rationality and validity of the presented FEM models are validated, and these models can precisely simulate the force-displacement response.

#### 3.2.2. Effect of IFs on Stiffness

**Figure 5:**
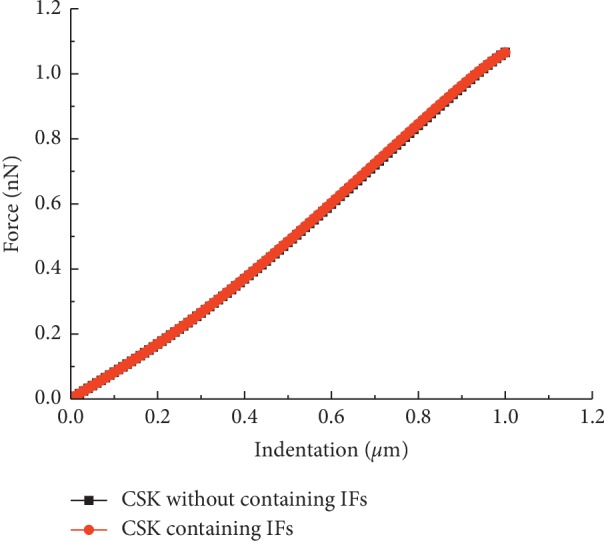
Force-indentation curves with two FEM models of a single cell.

It is widely accepted that IFs have a key role in cell mechanics [15]. The effect of IFs on overall cellular stiffness within the small deformation state was investigated. The results showed that IFs contribute only slightly to the overall cellular stiffness, as shown in Figure 5, which was consistent with the published conclusions [20, 21].

**Figure 6:**
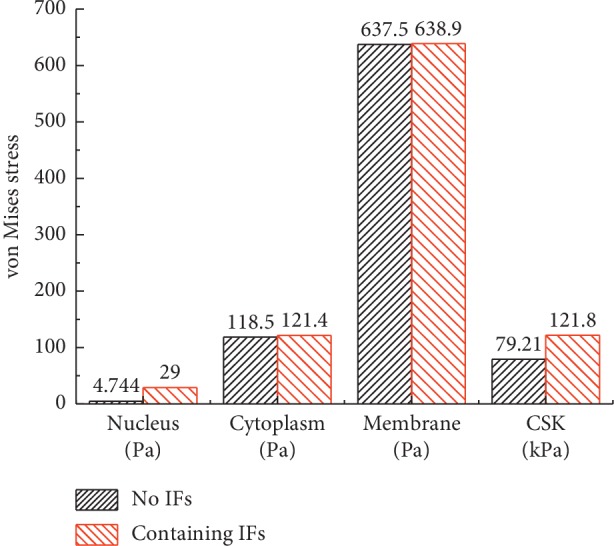
The maximum von Mises stress results of the cell components with two single-cell FEM models.

The maximum von Mises stress results of the major components of the cell tended to increase when IFs were included, as shown in Figure 6; however, the von Mises stress of the nucleus increased most significantly. The reasons for this results may be that the IFs can transfer forces directly from the membrane to the nucleus [25].

In addition, the maximum von Mises stress results of the CSK are 79.21 kPa and 121.8 kPa, respectively, while the maximum von Mises stress results of the continuum (membrane) are 0.6375 kPa and 0.6389 kPa, respectively. The former result is hundreds of times larger than the latter. The results indicate that the CSK is the major mechanical component of the cell, which is consistent with the findings of previous studies [14]. The results further prove that the CSK plays a key role in determining overall cellular stiffness and can support cell architecture and control cell motility.

#### 3.2.3. Parametric Variation in the Material Properties

**Figure 7:**
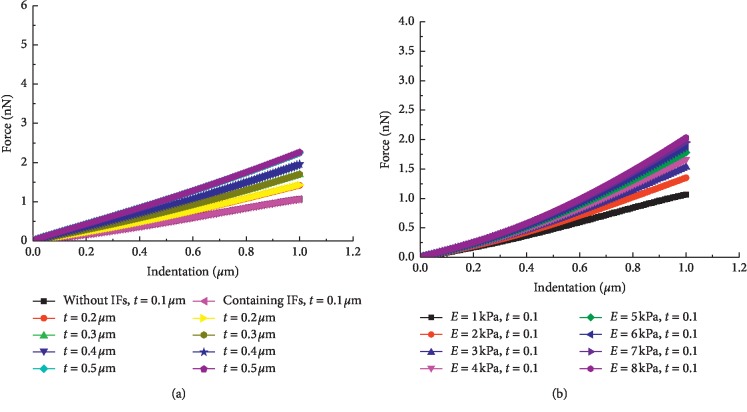
Force-indentation curves with different thicknesses and elasticities of the membrane. (a) Different thicknesses. (b) Different elasticities.

The influence of the membrane material properties on the overall cellular stiffness was studied by modulating the thickness and elasticity of the membrane. As demonstrated in Figure 7, the force-indentation curves tend to increase as the thickness or elasticity increases. The results show that the overall cellular stiffness increases with increasing thickness or elasticity of the membrane.

**Figure 8:**
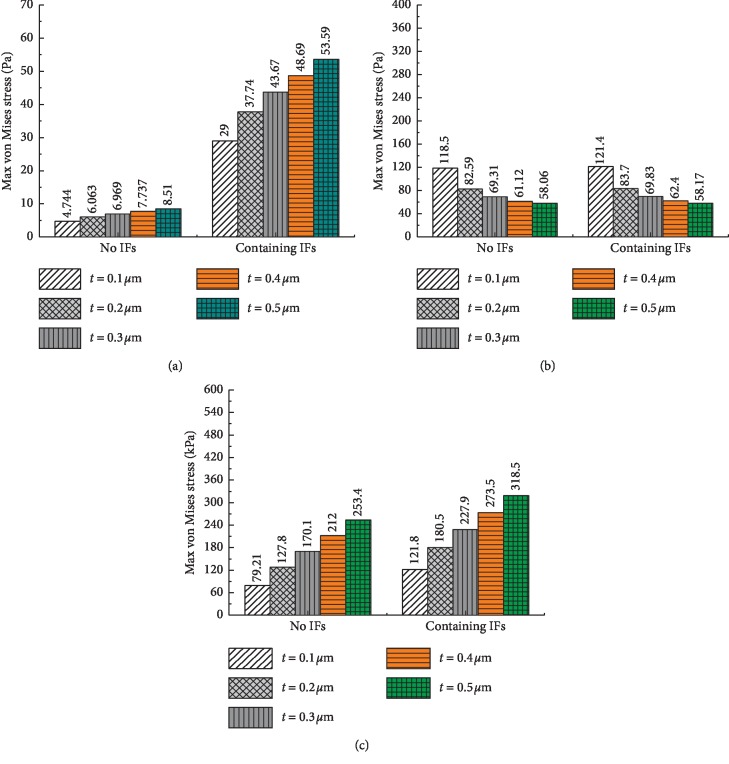
The maximum von Mises stress results of the cell components with different membrane thicknesses. (a) Nucleus. (b) Cytoplasm. (c) CSK.

**Figure 9:**
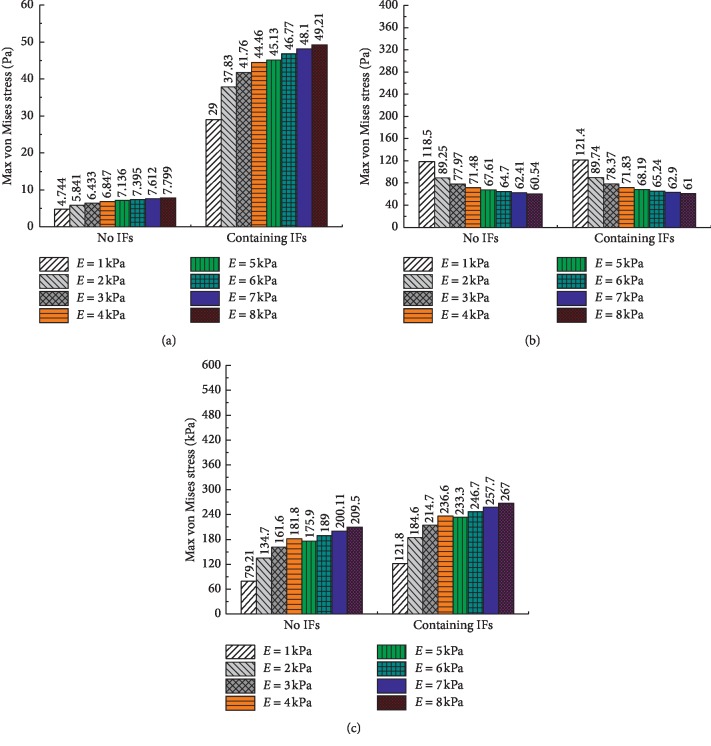
The maximum von Mises stress results of the cell components with different elasticities of the membrane. (a) Nucleus. (b) Cytoplasm. (c) CSK.

Additionally, the influences of the membrane thickness and elasticity on the cell components were investigated, as shown in Figures 8 and 9. The results show that the maximum von Mises stress results of the nucleus and CSK increase with increasing thickness and elasticity, while the maximum von Mises stress results of the cytoplasm decrease. The decrease in the results of the cytoplasm and the significant increase in the results of the CSK again prove that the CSK is the primary mechanical component of the cell.

**Figure 10:**
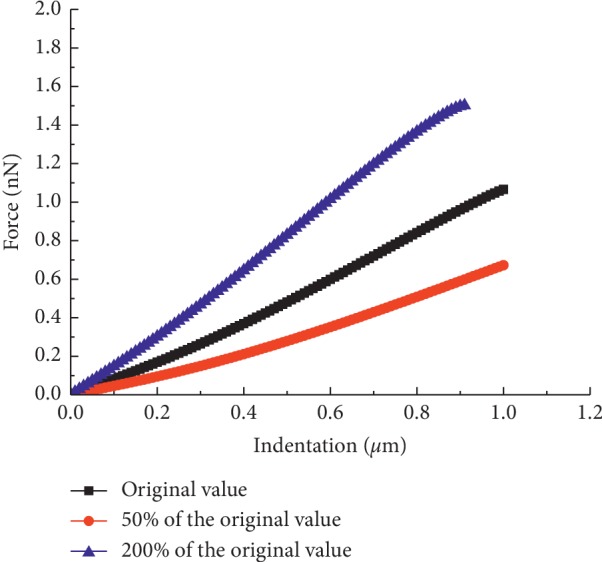
Force-indentation curves with different elasticities of the cytoplasm and nucleus.

Additionally, the influence of the cytoplasm and nucleus on the overall cellular stiffness was investigated by adjusting their elasticity to 50% and 200% of the original values, keeping the nucleus four times stiffer than the cytoplasm [14]. The elasticity of the cytoplasm and nucleus can cause an evident change in the overall stiffness, although their elasticity is less than that of the membrane (Figure 10). The reason for this result may be attributed to the large volume occupancy. The results showed that Young's modulus of the cytoplasm and nucleus has a prominent influence on overall cellular stiffness.

**Figure 11:**
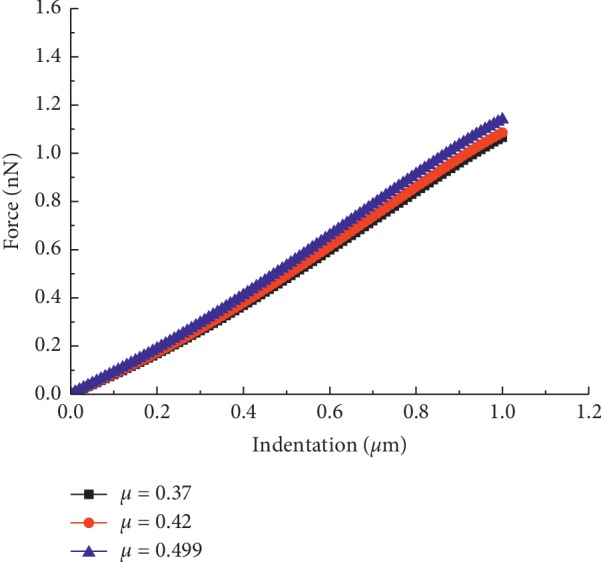
Force-indentation curves with different Poisson's ratios.

In the above studies, Poisson's ratio of the continuum was assumed to be 0.37 based on previous studies, which suggested that Poisson's ratio of living cells generally ranges from 0.35 to 0.5 [33]. However, theoretically, Poisson's ratio has a certain influence on simulation results, and three values (e.g., 0.37, 0.42, and 0.499) are chosen to verify the effect of Poisson's ratio and incompressibility of the continuum on the overall cellular stiffness. The force-indentation curves of different Poisson's ratios are shown in Figure 11. The results indicate that Poisson's ratio hardly affects the overall stiffness relative to other material properties. This result verifies that it is reasonable and feasible to choose Poisson's ratio of 0.37 in the FEM simulation.

## 4. Discussion

The results show that the force-indentation curves of the present FEM models match the AFM experimental data well. The FEM model simplifies the single cell as a hybrid elastic structure, combining continuum and tensegrity structural modelling. The AFM indentation experiments were conducted at room temperature, as Chiou et al. [34] reported that the differences in Young's modulus of NIH3T3 cells were not significant between body temperature and room temperature, and the apparent positional differences were observed. The FEM models treat the membrane, cytoplasm, and nucleus as linear elastic continua and the CSK as a tensegrity structure. The results indicate that IFs contribute only slightly to the overall cellular stiffness within approximately 10% strain (Figure 5), although the IFs can directly transfer forces from the membrane to the nucleus. Additionally, the sensitivity of the force indentation was studied by changing the material properties of the major cellular component, and the individual contributions of the major cell component to the overall cellular stiffness were identified.

Regretfully, this research has some limitations. An idealized geometry of the cell, instead of the actual geometry of the cell, was selected to conduct a parametric analysis of the material properties for a small deformation (<10%). The FEM models described here treat the CSK as a 12-node tensegrity structure and assume that the membrane, cytoplasm, and nucleus are linear elastic solids. This is obviously a considerable simplification because the cell components comprise intricate and dynamic viscoelastic networks of filaments, motors, and associated proteins. Moreover, the variation in the contact area between the indenter and the cell membrane was not considered during the indentation process. In fact, the contact area increased as the depth of the indenter into the cell increased. Therefore, the present FEM model can be further improved by including the aforementioned factors.

However, the FEM models presented here included enough valuable information to roughly predict the stiffness of the cell and evaluate the influence of the mechanical behaviour of cell components on the overall cellular stiffness.

## 5. Conclusions

In the present study, two new FEM models of a single cell were provided to account for the effect of IFs and the mechanical properties of the cell main components on the overall stiffness within 10% strain. The geometric sizes of the FEM models were obtained using AFM topography images. The rationality and validity of the FEM models were illustrated by comparing the experimental results at two different sites, and the error was approximately 10%. Thus, the proposed FEM models can be used to determine the influence of the major cell components on the overall cellular stiffness. Furthermore, this approach has a great potential to be implemented for cell intracellular force transduction and distribution simulation to promote a deeper understanding of mechanotransduction.

The main findings are summarized as follows:

- The experimental results suggested that the stiffness of cancer cells had apparent positional differences
- Although IFs can transfer forces directly from the membrane to the nucleus, they have little influence on the overall cellular stiffness for small deformations (within 10% strain)
- The material properties of a continuum (the thickness and elasticity of the membrane or Young's modulus of the cytoplasm and nucleus) have a prominent effect on the overall cellular stiffness, but Poisson's ratio has little influence compared with that of the other material properties
- CSK is the major mechanical component of cells and plays a key role in determining the overall stiffness of cells

## Figures and Tables

**Table 1:** Material parameters of cell components.

Materials	Elastic modulus (Pa)	Poisson's ratio (*v*)	Cross-sectional area (nm^2^)
Membrane [28, 29]	1000, 5000∼8000	0.3	Thickness = 0.1∼0.5 *μ*m
Cytoplasm [14]	100	0.37	∼
Nucleus [14]	400	0.37	∼
MTs [30]	1.2 × 10^9^	0.3	190
MFs [30]	2.6 × 10^9^	0.3	19
IFs [31]	2 × 10^9^	0.3	100

## Data Availability

The data used to support the findings of this study are included within the article.
